# Economic aspects of suppressing malaria in Africa

**DOI:** 10.5281/zenodo.10887781

**Published:** 2014-09-22

**Authors:** William R. Jobin

**Affiliations:** 1 Blue Nile Associates, 25558 Road N.6, Cortez, Colorado 81321, USA

## Abstract

**Background:**

Suppressing malaria in Africa is costly, but is it a good way for international agencies to use their funds, or alternatively, for the African nations that are the direct beneficiaries? Unfortunately, the current ephemeral methods in the malaria strategy of the World Health Organization have required continuous and rising expenditures by international donors who were beginning to lose interest by 2010. To avoid becoming hostage to international economic limitations, African countries might want to consider suppressing malaria themselves, and might want to add permanent and lasting methods to the WHO strategy. The purpose of this study was to determine whether investments in suppressing malaria might produce significant benefits for African nations.

**Materials and Methods:**

Two epidemiologic analyses were used in parallel to evaluate data from Africa: a before-after comparison of countries treated under the US President’s Malaria Initiative for Africa (PMI), and a simultaneous comparison of treated-untreated countries.

**Results:**

From 2007 to 2012, relative increases in population and gross domestic product (GDP) were greater in 14 countries treated as part of PMI than in 9 similar, but untreated countries. In the treated countries the relative increase in the GDP of 0.61 before malaria suppression rose to 0.64 afterwards; whereas in the untreated countries it fell from 0.67 to 0.56. The increase in GDP in the 14 treated countries that was attributable to malaria suppression over the 5-year interval was about $4.77 billion. During that period, the mean cost of suppressing malaria had been about $1.43 billion, indicating a return on the investment of 3.4 to 1. However, the costs began rising steeply in 2012.

**Conclusions:**

Malaria suppression might be worthwhile for African countries to undertake themselves, as long as the biocides and drugs in current use remain effective.

## 1 Introduction

In the early years of this century, the strategy for suppressing malaria in Africa, as recommended by the World Health Organization (WHO) and used by the US President’s Malaria Initiative for Africa (PMI), involved never-ending and rising expenses. The strategy was based on temporary measures requiring continual purchases of bed-nets, drugs and biocides. Thus, there was a need to evaluate the costs and economic returns as well as the public health benefits from such large and unending expenditures.

The methods used in the WHO strategy ‘suppressed’ malaria during the period under study, in the sense that they did not eliminate or eradicate it, but merely held it down temporarily. If the WHO methods were to be relaxed, transmission would rise. Unfortunately, the methods included no permanent alterations to ecology or epidemiology, which likely could have permanently reduced transmission.

Previous international programs to suppress malaria in Africa in the twentieth Century ended in failure for a variety of technical and financial reasons [1]. The most notable failure was the Global Malaria Eradication Programme (GMEP), started by the WHO in the 1950s with financial support from the US government and others. This poorly conceived programme collapsed in 1969, and evaluation of its costs and impacts was difficult. However, in 2005 the PMI began **–** another large-scale attempt to suppress malaria. Fortunately, it also became a valuable source of data for evaluating the impacts of malaria suppression [2,3]. It began in Angola and was expanded to treat 19 African countries by 2012, following a uniform global strategy recommended by WHO. The 19 countries were added gradually, and covered four of the major ecologic zones of Africa (Table 1).

**Table 1.** Nineteen countries in the PMI in 2012, located in four of the major ecological zones of Africa, showing the fiscal year* in which operations began

Country treated	Ecological zone	Fiscal year started
Angola	Savannah	2005
Tanzania	Grassland	2006
Uganda	Savannah	2006
Benin	Coastal Savannah	2007
Ethiopia	Grassland	2007
Ghana	Coastal Savannah	2007
Kenya	Grassland	2007
Liberia	Coastal Savannah	2007
Malawi	Savannah	2007
Mali	Grassland	2007
Mozambique	Savannah	2007
Rwanda	Savannah	2007
Senegal	Savannah	2007
Zambia	Savannah	2007
Madagascar	Grassland	2008
Congo-Kinshasa	Forest	2010
Nigeria	Coastal Savannah	2010
Guinea-Conakry	Savannah	2011
Zimbabwe	Savannah	2011

* In the US government, each fiscal year begins on 1 October

### 1.1 The ‘resistance treadmill’

The previous GMEP and other attempts at malaria suppression in Africa failed due to recurring resistance to biocides by the mosquitoes, and to drug resistance developed by malaria parasites [1,4]. Resistance to each new biocide developed so rapidly that malaria programmes seemed to be on a ‘resistance treadmill’. Some countries such as Sudan went through several major classes of biocides before their programmes collapsed [1]. Thus, it has been recommended that future attempts to suppress malaria should add more durable methods, which permanently change mosquito and human ecology, and thus reduce the pressure toward chemical resistance [5]. These methods should include improvements such as agricultural reclamation of swampy land where mosquitoes breed, and also improvements in housing, including metallic screens to protect people from mosquito biting. The strategies should be tailored to the specific epidemiology of each major ecologic zone, to improve their cost-effectiveness (Table l).

### 1.2 The ‘immunity trap’

There is an important immunological problem related to suppressing malaria in Africa by the ephemeral strategy of the WHO and PMI, especially because it requires sustaining expenditures of millions of US dollars. An important risk with this ephemeral strategy is that if financing – and thus the suppression – were to fail, the previously protect-ed populations would be caught in an ‘immunity trap’. After a few years of successful suppression of transmission, young children in the protected populations would not have been developing their acquired immunity to malaria. When transmission resumed – even for a short time – they would be at risk of severe disease and death [1].

### 1.3 Economic analyses

In a series of analytical studies, it has been demonstrated that malaria was related to low per capita income in the tropics, and that suppressing malaria should be conducted with careful attention to ecology [6].

In most analyses of the impact on productivity of sup-pressing malaria, the measure used to assess economic productivity is per capita gross domestic product, expressed as per capita GDP in terms of current US dollars and mid-year national populations [7]. As used here, per capita GDP was the aggregate output of all goods and services in a country, including personal consumption, government expenditures, private investment and capital and net exports, all divided by the size of the population.

An early estimate of the profitability of malaria suppression had been made from data from the copper mining belt in Zambia [8]. It was concluded that integrated malaria control in these mining communities had been a sound investment because it raised labour productivity. A controlled statistical analysis showed that in 1955 the incomes of people in tropical countries with malaria were only one-third the value of the incomes of people in countries with-out malaria. It was also found that a 10% reduction in malaria was associated with 0.3% higher GDP growth, a small but positive relation [9].

Based on a theoretical exercise involving expected disability and lives lost from malaria, it was estimated that if malaria were eliminated from Africa by fully funding the WHO strategy, there would result a $30 increase in per capita GDP [10]. In 2012 the mean per capita GDP values in malaria-endemic countries in Africa were about $500 [11]. An increase of $30 would be a small but positive benefit. Another theoretical exercise had estimated that funding malaria suppression in accord with global targets would have a ‘2013 net present value’ of $209 billion globally, after subtracting the cost of malaria suppression [12]. In 2013, most African countries with endemic malaria had GDPs over $20 billion, and more than $20 million was being spent annually in each country to suppress malaria [13]. An analysis of data on malaria, demography and per capita income in the Americas indicated that populations born after implementation of anti-malaria programmes had higher incomes than did preceding generations [14].

### 1.4 The US President’s Malaria Initiative for Africa (PMI)

Since its beginning the PMI had solid support from the US government, with an annual budget starting at $4 million in Fiscal Year (FY) 2005, rising to $604 million by FY 2012 [3]. The strategy used in the PMI followed the recommendations of WHO. Unfortunately, WHO recommended the same strategy throughout the tropical world, with little recognition of variations in local ecology and epidemiology. They also seemed unaware that the high and increasing cost of this strategy means that for its longterm success, economics might become more important than epidemiology.

Although there has been a recommendation by WHO for modification of routine chemotherapy procedures for the highly seasonal transmission patterns in countries of the Sahel Zone, in general their strategy of drugs, bednets and biocides is applied uniformly throughout Africa, with little recognition of durable actions, such as screening of houses, of elimination or management of larval habitats, or of other permanent improvements which lower the background intensity of transmission.

Epidemiological monitoring of PMI was guided by the Centres for Disease Control (CDC) of the US Public Health Service. Although detailed reports have been given on the effort and money expended to suppress malaria, evaluation of its impact on malaria transmission by CDC has been cursory. Once PMI got underway, the measure of impact reported was the decrease of deaths among children from all causes. However, there are problems with identifying which deaths were from malaria, among the ‘all-cause’ deaths of children. There are many causes of deaths among children, and many programmes in Africa working simultaneously to prevent these deaths;; the PMI being only one of them [15,16]. Because of the lack of solid epidemiological monitoring of PMI, we decided to base our analysis on the economic impact.

## 2 Materials and Methods

The purpose of this analysis was to estimate the economic impact of malaria suppression in African countries covered by PMI, in relation to expenditures. Two factors for which data were widely available for Africa were evaluated: population size and economic productivity. The classic techniques of comparing similar treated-untreated populations, along with before-after comparisons, were used in parallel to clarify the impact of malaria suppression.

Maximum similarity of the treated and untreated countries used in this analysis was sought by selecting African countries with similar situations [11].

All 54 African countries were separated into a treated group and an untreated group, selecting those which were similar in malaria experience, climate, biology, geography, political history and economy. The selection process yield-ed 14 countries for the treated group and nine countries in the untreated group [11].

Countries with at least 5 years of peaceful political history were included. Thus, all countries wracked by warfare during the previous 5 years were eliminated. Countries were selected which had economies based primarily on subsistence agriculture.

Countries whose economies included oil and mineral extraction and exportation were discarded, because an important source of variance in economic performance is the unusually high GDPs of these countries due to their ability to extract and export oil and precious minerals. Because of its large oil fields and diamond mines, Angola had a calculated per capita GDP over $5,000 in 2013, ten times that of Tanzania which depended on subsistence agriculture, but which had few valuable exports [13]. The economics of oil and mineral exportation are largely dependent on their prices on the world market and thus independent of labour efficiency, which might be affected by malaria. Clearly, the GDP data for oil- and mineral-exporting countries requires special treatment in a continental analysis, and were thus not included in our study.

A parallel evaluation was based on comparisons of these countries before and after treatment. This second evaluation was conducted to increase the certainty that the impact on population and economic productivity was due to the suppression of malaria and not due to global trends or because of important initial differences in the two groups of countries.

Available data were assembled on these countries for the years 2000 to 2012, including the 5-year interval before suppression of malaria began (2000-2005) and the 5-year interval after suppression had begun in all of the countries (2007-2012).

## 3 Results

Analyses using the two comparisons indicated a dramatic impact from malaria suppression, both in saving lives and in increasing economic productivity.

### 3.1 Saving lives

For the 14 treated countries, beginning with a total population of 265 million in 2000, the increase in population during the five years before PMI started was 31 million people, a relative increase of 0.12 (Table 2). However, in the five years after PMI started **–** from 2007 to 2012 **–** the population grew more rapidly, by 47 million, a larger relative increase of 0.15. In contrast, in the nine untreated countries, beginning with a total population of 50 million in 2000, the increase in population in the five years before PMI started was 8 million, a relative increase of 0.16. However, in the five years after PMI started **–** from 2007 to 2012 **–** the population grew more slowly, by only 7 mil-lion, a noticeably smaller relative increase of 0.11.

**Table 2.** Comparison of populations (in millions), for 14 treated and nine similar, but untreated countries, before and after PMI operations were launched

	Population before PMI				Population after PMI			
	Initial	Final	Change	Relative change*	Initial	Final	Change	Relative change*
	2000	2005			2007	2012		
14 treated countries	265	296	31	0.12	317	364	47	0.15
9 untreated countries	50	58	8	0.16	63	70	7	0.11

* Affected slightly by rounding of population data to nearest million

Population changes are determined by births, deaths and migration, none of which are measured consistently or uniformly across the large number of African countries. Variability's in birth rates are complex and include the effects of education of mothers, poverty or economic development and other socio-economic factors. Although an important cause of death in Africa, malaria is only one of many highly variable causes. Because of their complexity and the unreliability of available data, these complex factors were not treated separately in our analyses. Instead, we relied on the more reliable World Bank reports of national populations and national GDPs, as well as the simultaneous use of two classical statistical techniques for large groupings, in which we compared treated-untreated conditions, as well as before-after comparisons, to separate the impact of malaria suppression from other background factors.

Admittedly, it would have been better to compare the impact of malaria suppression on the actual number of deaths due to malaria. However, these numbers have not been measured directly. Although the numbers of malaria deaths have been estimated using data for all-cause death reports from national registries, massaged by complex computer manipulations, the unreliability of national registries, plus the uncertainty in the computer simulations, detracted from our confidence in these numbers. Thus, only total population numbers from national censuses were used in the analyses. They are the most reliable demo-graphic figures available for Africa.

### 3.2 Increases in GDP

In the 14 treated countries with a total initial GDP of 67 billion US dollars in 2000, during the five years before intervention, the increase in GDP was $40 billion, a relative increase of 0.61 (Table 3). In the five years after intervention, the increase in GDP was $101 billion, a larger relative increase of 0.64. In the nine untreated countries, beginning with a total initial GDP of $12 billion, the increase in GDP was $8 billion, a relative increase of 0.67. For the final period, the increase in GDP was $14 billion, a smaller relative increase of 0.56.

**Table 3.** Comparison of economic productivity, as GDP (in billions of US dollars), for 14 treated and nine untreated, but similar countries, before and after PMI operations were launched*

	GDP before PMI				GDP after PMI			
	Initial	Final	Change	Relative change**	Initial	Final	Change	Relative change**
	2000	2005			2007	2012		
14 treated countries	67	107	40	0.61	159	260	101	0.64
9 untreated countries	12	20	8	0.67	25	39	14	0.56

* Per capita gross domestic product GDP is expressed in terms of the current US dollars and mid-year national populations [7]

** Affected slightly by rounding of population data to nearest million

Combined, the relative increases in population and GDP were greater in the treated countries than in the untreated ones. In the treated countries the relative population increase of 0.12 before malaria suppression rose to 0.15 afterwards, whereas in the untreated countries the relative population increase fell from an initial 0.16 to 0.11, quite the reverse of the population trends experienced in the treated countries. Use of the two epidemiologic comparisons together indicated that relative increases in population in the treated countries probably occurred due to the malaria suppression, although changes in birth and migration rates might also have been involved.

The economic similarity of the countries in the treated and in the untreated groups was confirmed by their similar per capita GDPs in the 5-year period before intervention began (Table 4). In 2000 the per capita GDP was $253 in the treated countries and $247 in the untreated countries. Again in 2005 – just before the PMI began – it was $360 in the treated countries and $338 in the untreated countries. The trends in GDP related to the PMI showed a pattern similar to the trends in population. In the treated countries the relative increase of 0.61 before malaria suppression rose to 0.64 afterwards, whereas in the untreated countries it fell from 0.67 to 0.56 (Table 3).

**Table 4.** Comparison of per capita economic productivity (in US dollars), for treated and untreated countries before and after PMI operations were launched*

	Per Capita GDP before PMI				Per Capita GDP after PMI			
	Initial	Final	Change	Relative change**	Initial	Final	Change	Relative change**
	2000	2005			2007	2012		
14 treated countries	253	360	107	0.42	502	713	211	0.42
9 untreated countries	247	338	91	0.37	404	560	156	0.39

* Per capita gross domestic product GDP is expressed in terms of the current US dollars and mid-year national populations [7]

** Affected slightly by rounding of population data to nearest million

In the face of the current complexity of changes in population and GDP in Africa, it is important to point out that we deliberately used two classical statistical methodologies simultaneously in our comparisons. First, we compared treated countries (countries in which PMI was suppressing malaria) with untreated countries (where PMI was not active). Secondly, we compared data from the treated countries before and after intervention (treatment) by PMI. By running these comparisons simultaneously for the two groups of countries, we minimised the effects of extraneous factors. We could not eliminate those factors, but using the two techniques simultaneously, we did minimise them.

### 3.3 Perverse effect of population changes

Although saving lives due to the decrease in malaria was an important public health accomplishment, it had a perverse effect on calculations of per capita economic productivity.

In the treated countries, the per capita GDP rose by $107 before malaria suppression and rose by $211 afterwards (Table 4). This was roughly the same relative change (0.42) during both periods, because the increased productivity was offset by increased population due to fewer malaria deaths.

In the untreated countries the per capita GDP rose by $91 in the five years before malaria suppression but rose by $156 afterwards. This was an increase in the relative change (from 0.37 to 0.39) because of the slow rise in their populations (Table 2). This picture was further complicated because the improved productivity due to less malaria took place in the adult work force, whereas the population increase – at least in the short term – was in young children.

Although it is true that the bednet and chemotherapy programmes were focused on children and would primarily prevent childhood deaths, the indoor spraying benefitted adults as well, preventing repeated infections and the debilitating attacks suffered by semi-immune adults. Thus, adult labour productivity would improve, showing as an increase in economic productivity for these adults, who were largely subsistence farmers. Nonetheless, in absolute terms, the increase in per capita GDP in the treated countries was $211, substantially higher than the increase of $156 in the untreated countries, indicating an important economic benefit from the malaria suppression.

To estimate the amount of investment, the mean annual expenditure of PMI during the five years after all 14 countries were in the programme was calculated [11]. This was an annual mean of $286 million for the 5-year period; an average of $135.7 million in 2007 and $436.95 million in 2012 (Table 5). The mean expenditures in the 14 countries were thus about $1.20 per capita in 2012, compared with the mean during the early years of PMI of only $0.43 per capita [13].

**Table 5.** Actual reported per capita expenditures in US dollars by PMI in 14 treated countries, 2007-2012

Fiscal year	2007	2012
Global expenditures (million $)	154.2	603.7
Expenditures in other countries (million $)	18.5	166.75
Expenditures in 14 countries (million $)	135.7	436.95
Population in 14 countries (million)	317	364
Mean annual expenditures per capita (in $)	0.43	1.20

Calculation of the return on investment was made by assuming that the relative increase in GDP over the 5-year interval was the mean of the combined values for the treated countries before suppression plus the values for the untreated countries before and after suppression, a relative increase in GDP of 0.61 (Table 3). After malaria was suppressed, the relative increase in the PMI countries over the five years rose to 0.64, thus the relative increase due to malaria suppression was 0.64-0.61 or 0.03. The initial GDP of the 14 untreated countries was $159 billion, thus the increase due to malaria suppression for the 5-year interval was $159 billion × 0.03 = $4.77 billion. The total expenditures of PMI for these 14 countries during this 5-year interval was calculated by multiplying the mean of annual expenditures for 2007-2012 of $286 million, times five years, which yielded $1.43 billion. Comparing it with the increased GDP of $4.8 billion indicated a return on PMI expenditures of 3.4 to 1 due to malaria suppression.

Unfortunately, there had been a threefold increase in per capita costs of the Initiative between 2007 and 2012 (Table 5). Because population coverage was not being increased, this was most likely due to resistance problems, and was accompanied by a loss of effectiveness of the applied pyrethroid insecticide in Tanzania and in West Africa, and subsequent loss in the effectiveness of the treated bednets [17]. Also, resistance to ACT, the drug of choice, has appeared in Southeast Asia and will probably surface soon in Africa, following the same geographical path as had resistance to chloroquine in the 1960s [17]. The sharply rising costs and decreasing effectiveness of the strategy indicated the increasing risk by 2013 of the collapse of the PMI, for the same reasons that the GMEP collapsed in 1969.

Although 40 years have elapsed since the collapse of the GMEP – and many conditions have changed, including the drugs and biocides used, the state of the African economies, and our knowledge about malaria – one important weakness has not changed: the use of what we call the ‘specialist approach‘, because the only methods employed were those familiar to physicians and public health specialists.

In recent reports, WHO has seemed overly optimistic with regard to its campaign against this deadly and debilitating disease. In fact, they are facing some serious fundamental problems. A major danger is that of repeating the disaster of the organization's GMEP that began in euphoria in 1955 but ended in disillusionment in 1969. It collapsed in large part because it adopted a one-size-fits-all reliance on limited tools, spraying with the seemingly all-powerful insecticide DDT, and provision of the inexpensive and initially effective drug, chloroquine [F. Snowden, *pers. comm.*]. The intervention was devised as a ‘quick fix’ rather than a sustained and flexible campaign against an intractable and ever-evolving foe. When mosquitoes developed resistance to the insecticide, and the malaria parasites developed resistance to medication, the programme was doomed to fail. The failure was all the more bitter because of the profoundly misleading promises of rapid eradication with which the campaign was launched.

Current WHO programmes are worryingly similar. The previous GMEP had adopted a ‘specialist approach’, and the WHO programme today relies on similar specialist, short-term methodology. Once again the antimalarial campaigners spray the inside of houses with insecticides that are powerful and dramatically effective – but only in the short term. They distribute temporary bednets treated with the same ephemeral biocides, and they administer drugs that save lives – again only in the short term, without altering the basic ecology of malaria transmission, thereby leaving people vulnerable to rebound epidemics in the future. Furthermore, these methods are costly because they must be continually reapplied. Annual expenditures on suppressing malaria exceeded $2 billion by 2010, with only partial global coverage [18].

## 4 Discussion

The author had previously evaluated data from the four initial years of the PMI in a correlation-regression analysis, which showed that the increase in economic productivity was proportional to the expenditures on malaria suppression, suggesting a causal relation [13]. This analysis of the annual per capita expenditures of PMI and the annual increases in per capita GDP for 14 countries during the 4-year interval from 2007 to 2011 showed that the slope of the regression line indicated a return on investment greater than 6.5:1.

The author’s second economic analysis of the countries in the PMI compared the economic impact in 14 countries with nine similar, but untreated countries for the 7-year period from 2005 to 2012 [11]. In this second study, only the treated-untreated comparison was made, without the before-after comparison included in this article. Perhaps this explains why the calculated return on investment was so high in the previous study. The author corrected the mean per capita expenditure of $0.66 per year from Table 1 [11], to $0.81 per year, based on a more accurate revision of data from PMI. However, the original $132 per capita GDP benefit over the 7-year period was used, as originally reported in Table 1 and 2 [11]. This corrected calculation resulted in an estimated return on investment for the 7-year period of $132/$5.67 or a ratio of 23.3:1. This high rate of return on investment was probably due to the influence of the general increases occurring for GDP in all of Africa. This influence was eliminated only when the before-after comparison was included in the current analysis. Perhaps the lower return of 3.4:1 from this analysis covering the 5-year interval was also due to the escalating costs after 2010 [3].

The author’s three analyses of data on GDP and PMI expenditures were each for slightly different years, but they all indicated that the return on investment in malaria suppression was quite high. Estimated returns for investments in suppressing malaria thus ranged from 3.4 (this study) to 6.5 [13] to 23.3 [11], depending on the type of analysis and the years evaluated.

To maximise cost-effectiveness it is important that national strategies be tailored to the local ecology [4]. Previous investigators also noted the importance of malaria ecology in the history of malaria in Africa [17]. When comparing the detrimental effects on economic development in Africa from the slave trade, from colonial exploitation and from the ecology of malaria, it was concluded that the most important factor was the ecology of malaria [19].

The perverse impact of lower death rates is important in evaluating the impact of malaria suppression when calculating per capita economic productivity. Contrary to most findings on the subject, a complex computer estimation of malaria deaths and disease – including extrapolations about impacts on economic consequences – led to the surprising conclusion that it was not economically profitable to fight malaria. Ashraf *et al.* [20] identified the importance of perverse population increases due to malaria suppression, when calculating per capita changes in GDP (Table 4). However, because of their myriad assumptions, coefficients and extrapolations inherent in such complex simulations, the simulations should be compared with reality before basing any policy decisions on their predictions [14,20].

Although the overall global funding for malaria control has grown recently, this is due entirely to the steadily increasing funding from the US for their PMI. UN financial sources for the Global Fund have not shown the same strength, and their recent poor performance has been noted [21]. The malaria funding crisis of 2010 occurred because cost of the WHO strategy had begun to rise rapidly, and effectiveness began to drop as the mosquitoes became resistant to the cheap and relatively safe pyrethroid biocides. This required the use of other more expensive and dangerous biocides for indoor spraying and also reduced effectiveness of the pyrethroid-treated bednets [17]. The funding crisis for malaria suppression occurring in the Global Fund was seen by some as a window of opportunity for African countries to invest more in their own health [18,22]. With the knowledge that investments in malaria suppression carry a remarkable return on investments of 3.4:1 or greater, there is surely financial justification for this view [23].

Escaping reliance on external donors and WHO policies would also give African countries more flexibility in attacking malaria by methods more suitable to their individual ecology and local situations. Durable methods, which could be added to the ephemeral WHO methods, include housing improvements to keep mosquitoes out with metallic screens on doors and windows, and filling of mosquito-breeding swamps. Swamp reclamation for agricultural purposes would also be an additional source of increased agricultural productivity to feed the growing populations. Perhaps the greatest and most direct benefit of malaria suppression in Africa is to increase the productivity of subsistence farmers, thus making it possible for them to feed the growing number of their surviving children, while also increasing national productivity.

## 5 Conclusions

From simultaneous evaluations based on treated-untreated comparisons and on before-after comparisons, relative increases in population and GDP were found to be greater in 14 African countries treated under the US Presidential Malaria Initiative from 2007 to 2012 than in nine comparable untreated countries.

When adjusted for population changes, the increases for the 14 treated countries in per capita GDP of $107 during the 5-year interval before suppression rose to $211 during the 5-year interval after suppression, whereas in the nine untreated countries it rose from $91 to only $156 over the same time intervals. Findings from these data con-firmed results from a previous correlation-regression analysis which showed that malaria suppression was a highly profitable investment for Africa. Estimates of return on investment thus ranged from 3.4:1 to 6.5:1, to as high as 23.3:1, depending on the years analysed and the method of analysis.

It is important also to recognise that the benefits will accrue primarily to the people protected, and will show up in improved health and survival, and in increased labour output of adults. However, if the funding for malaria suppression were from international agencies, they would not experience these direct benefits, although they might be rewarded in other ways. If the funding is from national sources, they will experience the benefit of increased GDP, as well as the improved health of their citizens. Finally, it is important to recognise that these favourable conditions might not endure much longer, because of growing resistance in Africa to pyrethroid insecticides, and even resistance by the malaria parasites to the current drug of choice used in the global WHO strategy.
